# Fabrication of Liquid-Contact Polymeric Membrane Ion-Selective Microelectrodes with a Long Lifetime

**DOI:** 10.3390/membranes16070216

**Published:** 2026-06-26

**Authors:** Yuanxin Liu, Longbin Qi, Wei Qin

**Affiliations:** 1 Shandong Key Laboratory of Coastal Zone Environmental Processes and Ecological Security, Yantai Institute of Coastal Zone Research, Chinese Academy of Sciences, Yantai 264003, China; liuyuanxin23@mails.ucas.ac.cn; 2 University of Chinese Academy of Sciences, Beijing 100049, China; 3 Laboratory for Marine Biology and Biotechnology, Qingdao Marine Science and Technology Center, Qingdao 266237, China; 4 Center for Ocean Mega-Science, Chinese Academy of Sciences, Qingdao 266071, China

**Keywords:** potentiometry, ion-selective microelectrodes, lifetime, electrode fabrication

## Abstract

The lifetime of polymeric membrane ion-selective microelectrodes is one of the key factors limiting their practical applications. Herein, we report a simple strategy for the fabrication of liquid-contact ion-selective microelectrodes with a long lifetime by increasing the thickness of the polymeric sensing membrane via filling the membrane solution from the back of the micropipette tip. A lead ion-selective microelectrode (Pb^2+^-ISμE) was used as a model sensor. The geometry of the electrode tip and the amount of membrane solution were optimized. The microelectrode exhibited a Nernstian response slope of 27.4 ± 0.7 mV decade^−1^ over the concentration range from 1.0 × 10^−7^ to 1.0 × 10^−3^ M with a detection limit of 4.2 × 10^−8^ M. Notably, the response slope of the microelectrode decreased by only 2.2% in 30 days.

## 1. Introduction

Polymeric membrane ion-selective electrodes (ISEs) have become a powerful analytical tool in clinical analysis and environmental monitoring owing to their attractive characteristics, such as high selectivity, low cost, and ease of operation [1,2]. With the significant need for in situ analysis of samples with a small volume and the development of micro/nano fabrication technologies, polymeric membrane ISEs are developed from the macroscale to the micro- and even nanoscale [3]. Nowadays, polymeric membrane ion-selective microelectrodes play important roles in many fields, such as single-cell detection [4,5,6], brain science research [7,8,9], plant physiology study [10,11], and sediment analysis [12,13,14].

Generally, ion-selective microelectrodes can be divided into two categories based on their configurations: liquid-contact and solid-contact microelectrodes [15]. Liquid-contact microelectrodes consist of an ion-selective membrane (ISM), an internal reference electrode and an internal filling solution. For solid-contact microelectrodes, they are composed of an ISM, a solid contact (SC) layer and an electronic conduction substrate [16]. The SC layer formed between the ISM and the electronic conduction substrate is essential for fabricating stable microelectrodes. The intrinsic properties of SC materials significantly influence potential stability and long-term reproducibility [17]. The ISM is the most critical component of microelectrodes. Originally, an ISE based on a liquid membrane incorporating an ionophore was developed by Simon [18]. In recent decades, solvent-polymeric membranes with polymeric matrices (normally containing plasticizers and doped with ionophores and ion exchangers) have predominated over liquid membranes [15]. Among the various polymeric matrix materials, polyvinyl chloride (PVC) is the most widely used polymer. In addition, solid-state membranes based on crystalline materials (e.g., Ag_2_S/Ag and copper hexacyanoferrate) have also been used to fabricate ion-selective microelectrodes [19,20].

Lifetime is one of the critical parameters that limit the practical applications of microelectrodes. Generally, the lifetime of an electrode refers to the period during which the electrode can function properly while maintaining its performance [21]. When a microelectrode’s key quantitative performance parameters (e.g., response slope and detection limit) degrade, which results in unacceptable error, the measured results are no longer reliable for the intended application and the electrode can no longer be used. Microelectrodes reported in existing studies tend to have a short service life, typically a few days and, in some cases, only hours [12,13]. Factors such as loss of ionophore and other membrane components and physical damage to the membrane can affect the lifetime [22]. For SC microelectrodes, detachment of the membrane from the conductive substrate and the formation of a water layer between the ISM and the substrate can lead to a short lifetime [23,24]. These adverse effects could be more pronounced for microelectrodes with a thin ISM. To improve the robustness of polymeric membrane ISEs, researchers have developed various methods, for example, the covalent binding of ion-exchanger sites and ionophores to a polymer backbone [25,26] and the covalent attachment of ISE membranes to both polymeric electrode bodies and the underlying conductive substrate [23]. However, these strategies are not suitable for microelectrodes. The primary reason for this is the difficulty of performing multi-step chemical modifications within the confined geometry of the micropipette tip.

Herein, we proposed a simple and configuration-independent method to fabricate liquid-contact ion-selective microelectrodes with a long lifetime by increasing the thickness of the polymeric sensing membrane. The enhanced thickness not only improves the mechanical stability of the ISM but also provides a larger amount of membrane components, thereby mitigating the adverse effects associated with component leaching. A PVC-based polymeric membrane Pb^2+^-ISμE was used as a model. The geometry of the electrode tip and the volume of the membrane solution were optimized. The lifetime of the proposed microelectrode was also compared with that of microelectrodes reported in the literature.

## 2. Materials and Methods

### 2.1. Material and Chemicals

Lead ionophore IV [*tert*-butylcalix [4]arene-tetrakis(N,N-dimethylthioacetamide)], high-molecular-weight poly(vinyl chloride) (PVC) and 2-nitrophenyl octyl ether (*o*-NPOE) were purchased from Sigma-Aldrich (St. Louis, MO, USA). Sodium tetrakis 3,5-bis(trifluoromethyl)phenyl borate (NaTFPB) was purchased from Alfa Aesar. A glass capillary (o.d.: 1.5 mm, i.d.: 1.10 mm, length: 10 cm) was purchased from Sutter Instruments Co., Ltd. (Novato, CA, USA). Tetrahydrofuran (THF), N-Dimethyltrimethylsilylamine and sodium chloride (NaCl, 99.99%) were obtained from Aladdin Biochemical Technology Co., Ltd. (Shanghai, China). Other chemical reagents were obtained from Sinopharm Chemical Reagent Co., Ltd. (Shanghai, China) and were of analytical reagent grade. Deionized water (18.2 MΩ·cm) was obtained using a laboratory ultrapure water system (UPHW-I-90T, Ulupure, Chengdu, China).

### 2.2. Fabrication of the Pb^2+^-ISμE

The Pb^2+^-selective membrane contained 1.57 wt% lead ionophore IV, 0.48 wt% NaTFPB, 32.65 wt% PVC and 65.3 wt% *o*-NPOE [27]. In total, 360 mg of these components was dissolved in 3.6 mL of THF to obtain the membrane cocktail solution.

**Figure 1:**
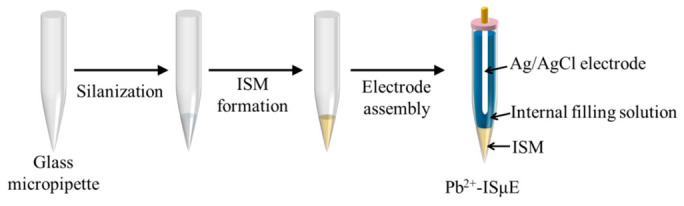
Schematic diagram of the fabrication process of the liquid-contact Pb^2+^-ISμE.

The fabrication process of the Pb^2+^-ISμE is shown in Figure 1. A glass micropipette was fabricated using a micropipette puller (P1000, Sutter Instruments, Novato, CA, USA). The micropipette tip was observed using an optical microscope before use. Micropipettes with a tip diameter of approximately 5 µm were selected. The micropipette tip was then immersed in N-(trimethylsilyl)dimethylamine for silanization at 150 °C for 3 h. Subsequently, the micropipette was backfilled with a 2.5 μL membrane cocktail solution. Positive pressure was applied to the back of the micropipette to achieve complete filling of the tip. After the THF in the membrane cocktail solution had evaporated for 3 days, a Pb^2+^-selective membrane was formed in the micropipette tip. A solution containing 1 mM Pb(NO_3_)_2_ and 1 mM NaCl was used as the internal filling solution. A Ag/AgCl electrode was inserted through the back end of the micropipette to make contact with the internal filling solution.

### 2.3. Characterization

Images of the microelectrode tip were captured using an optical microscope (CX31, Olympus, Tokyo, Japan). The thickness of the membrane was measured based on the scale bar in the microscope field of view. Electrochemical impedance spectroscopy (EIS) was performed using an electrochemical workstation (CHI660E, Chenhua, Shanghai, China). The Pb^2+^-ISμE served as the working electrode, a platinum wire electrode (CE-PT-W-0537, Gaossunion, Tianjin, China) served as the counter electrode, and a Ag/AgCl/3 M KCl electrode (RE-AgCl-1038, Gaossunion, Tianjin, China) served as the reference electrode in the three-electrode cell system. EIS spectra were obtained in the frequency range from 100 kHz to 0.01 Hz. The amplitude of the excitation signal was 100 mV. The experiments were performed in a 0.1 M KCl solution at open circuit potential.

### 2.4. Potentiometric Measurements

The potentiometric responses of microelectrodes were measured by using an electrochemical workstation, which had an input impedance of 10^12^ Ω. The Pb^2+^-ISμE was used as the indicator electrode. A double-junction electrode (Ag/AgCl/3 M KCl/1 M LiOAc) was used as the reference electrode. Prior to use, each Pb^2+^-ISμE was sequentially conditioned in 10^−3^ M Pb(NO_3_)_2_ for 2 days and 10^−5^ M Pb(NO_3_)_2_ for 2 days. The internal filling solution was replaced by 10^−5^ M Pb(NO_3_)_2_ with 10^−3^ M NaCl when the Pb^2+^-ISμE was immersed in 10^−5^ M Pb(NO_3_)_2_. Immersing electrodes in 10^−3^ M Pb(NO_3_)_2_ enables rapid conditioning of the electrode. Subsequently, the conditioning solution and internal solution were changed to 10^−5^ M Pb(NO_3_)_2_, which was intended to reduce the ion flux from the electrode membrane, thereby lowering the detection limit of the electrode. Selectivity measurements were performed using the Bakker’s modified separate solution method [28]. Before measurements, the electrodes were conditioned in 10^−3^ M NaCl solution overnight. The measured potential values were corrected for the liquid-junction potentials according to the Henderson equation, and the activities were calculated according to the Debye–Hückel equation. The detection limits of the ISEs were calculated according to the recommendations of the International Union of Pure and Applied Chemistry (IUPAC). The lower detection limit is the activity of the target ion at the intersection of the extrapolated linear portion and the low-level non-responsive portion of the calibration curve.

## 3. Results and Discussion

### 3.1. Optimization of Microelectrode Fabrication

The fabrication of ion-selective microelectrodes can be divided into two strategies. The first is filling of the membrane solution from the tip of the micropipette, generally achieved by capillary action or negative pressure aspiration. Alternatively, microelectrodes can be fabricated by dip-coating a tip in a membrane solution, where the tip is integrated with a conductive substrate. However, these methods yield a relatively thin ISM. The second strategy is backfilling of the membrane solution from the rear of the micropipette tip. To date, no research has been conducted to investigate the influence of membrane thickness on electrode tip integrity, such as the detachment of the membrane from the micropipette wall and the electrode lifetime. In this work, the back-filling approach was employed to optimize membrane thickness relative to micropipette tip geometry, thereby yielding microelectrodes with extended lifetimes.

**Figure 2:**
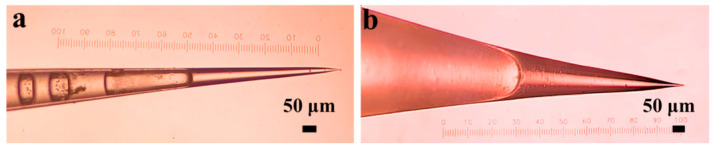
Images of micropipettes with a needle-shaped and tapered tip before (**a**) and after (**b**) filling with the internal solution and ISM.

The shape of the micropipette tip significantly affects the microelectrode fabrication process for liquid-contact microelectrodes. Micropipettes with different tip shapes (needle-shaped and tapered) were prepared by adjusting the parameters of the puller while keeping the diameter of the micropipette tip constant at 5 μm. The needle-shaped tip may cause high air resistance during backfilling of the internal filling solution, making it difficult to fully contact the ISM (Figure 2a). In contrast, the tapered tip allows easy contact between the filling solution and the membrane (Figure 2b). Therefore, the tapered tip micropipette was selected for subsequent microelectrode preparation.

**Figure 3:**
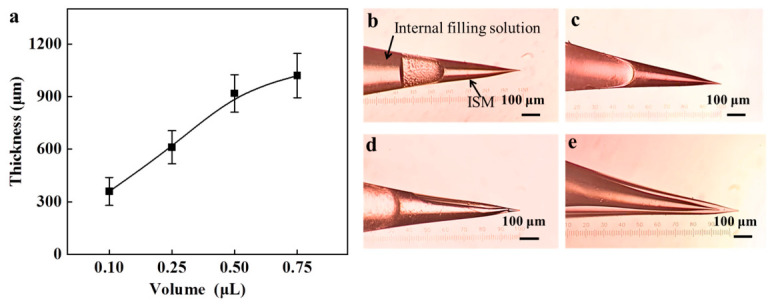
Influence of the volume of membrane solution on the thickness of ISM (**a**) and images of the tip of the Pb^2+^-ISμE obtained using cocktail solution volumes of 0.10 μL (**b**), 0.25 μL (**c**), 0.50 μL (**d**) and 0.75 μL (**e**).

The thickness of the ISM was also optimized by controlling the amount of membrane solution used. As shown in Figure 3a, an increase in membrane thickness was observed when the volume of the cocktail solution increased. It should be noted that 0.10 μL of cocktail solution produced a short membrane, which resulted in a failure of contact between the internal filling solution and the ISM (Figure 3b). When the membrane solution volume exceeded 0.25 μL, the internal filling solution readily contacted the ISM. However, at volumes greater than 0.5 μL, the ISMs tended to detach from the inner wall of the micropipettes due to the shrinkage of the membrane solution during the evaporation of THF (Figure 3d,e). Therefore, 0.25 μL was selected for microelectrode preparation, which produced an ISM of approximately 600 μm. The standard deviation of sensitivity for five independently prepared electrodes was 0.7 mV/deacde^−1^, indicating the good fabrication reproducibility of the proposed method. The resistance of the microelectrode was 3.6 GΩ, which is higher than that of solid-contact ion-selective microelectrodes (~10^6^ Ω) but comparable to that of liquid-contact ion-selective microelectrodes.

### 3.2. Potentiometric Performance

**Figure 4:**
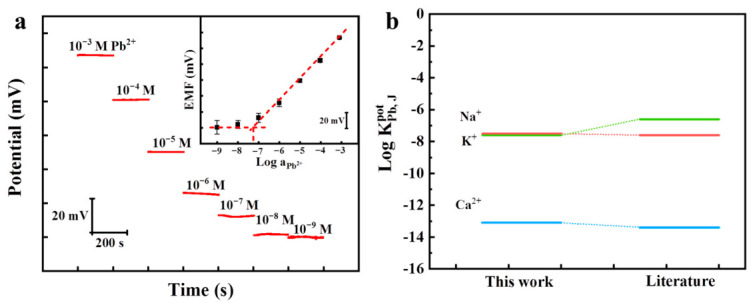
Typical response and calibration curve (insert) for the Pb^2+^-ISμE (**a**). Comparison of selectivity coefficients for the Pb^2+^-ISμE and those reported in the literature [12] (**b**). Error bars represent the standard deviation of three measurements.

The potentiometric response of the Pb^2+^-ISμE was investigated in Pb(NO_3_)_2_ solution over the concentration range of 1.0 × 10^−9^ to 1 × 10^−3^ M. As shown in Figure 4a, the Pb^2+^-ISμE exhibited a linear response in the concentration of 1.0 × 10^−7^–1.0 × 10^−3^ M with a Nernstian slope of 27.4 ± 0.7 mV/decade. The detection limit was calculated to be 4.2 × 10^−8^ M. The response time (t_95%_) was approximately 60 s. Such a slow response of the electrode was mainly due to the high impedance of the Pb^2+^-ISμE. Selectivity coefficients of the Pb^2+^-ISμE toward various interfering ions (i.e., Na^+^, K^+^ and Ca^2+^) were examined using Bakker’s modified separate solution method. As shown in Figure 4b, selectivity coefficients of the Pb^2+^-ISμE towards Na^+^, K^+^ and Ca^2+^ are −7.5, −7.6 and −12.9, respectively, which are in agreement with those of the ISE in the literature [12]. Therefore, the proposed Pb^2+^-ISμE shows good potential for environmental sample analysis.

### 3.3. Reproducibility and Stability

**Figure 5:**
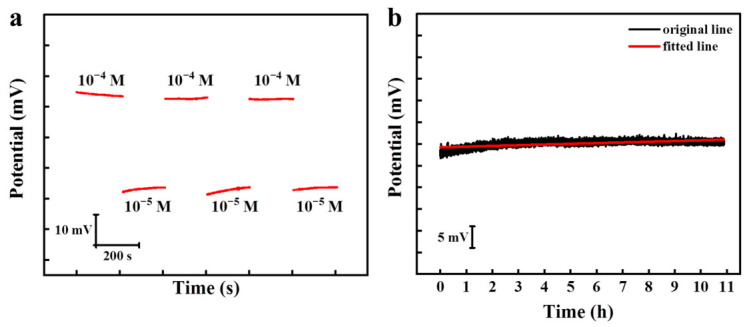
(**a**) Potential reproducibility of the Pb^2+^-ISμE evaluated by alternatively measuring 10^−5^ M and 10^−4^ M Pb(NO_3_)_2_ solutions without stirring. (**b**) Long-term stability of the Pb^2+^-ISμE in 10^−5^ M Pb(NO_3_)_2_ under continuous stirring (300 rpm).

The reproducibility of the Pb^2+^-ISμE was investigated by alternatively measuring 10^−5^ and 10^−4^ M Pb(NO_3_)_2_ (Figure 5a). The standard deviations of the potential values were calculated to be ±0.32 mV for 10^−5^ M Pb(NO_3_)_2_ and ±0.04 mV for 10^−4^ M Pb(NO_3_)_2_ (*n* = 4), respectively. Moreover, the long-term stability of the Pb^2+^-ISμE was tested for 10 h in 10^−5^ M Pb(NO_3_)_2_ solution under continuous stirring. As shown in Figure 5b, the black line represents the original data and the red line is a linear fit line to the original data, which is used to facilitate the calculation of the potential drift. The response stabilizes after an initial equilibration period. The potential drift of the Pb^2+^-ISμE was calculated to be 109 μV h^−1^. These results indicate that the Pb^2+^-ISμE exhibited good reproducibility and stability. Figure 5 also reveals the noise level of the electrode. In the absence of stirring, the noise level of the microelectrode was measured to be ±0.1 mV in 10^−5^ M Pb(NO_3_)_2_ (Figure 5a). Under stirring at 300 rpm, the noise level was calculated to be ±0.5 mV in 10^−5^ M Pb(NO_3_)_2_ (Figure 5b). This noise is probably attributable to the high electrode impedance arising from the increased thickness of the polymer membrane. Over the linear range of 10^−7^ to 10^−3^ M, the observed noise may lead to a relative error of ±4.2%, which is considered acceptable.

### 3.4. Lifetime

**Figure 6:**
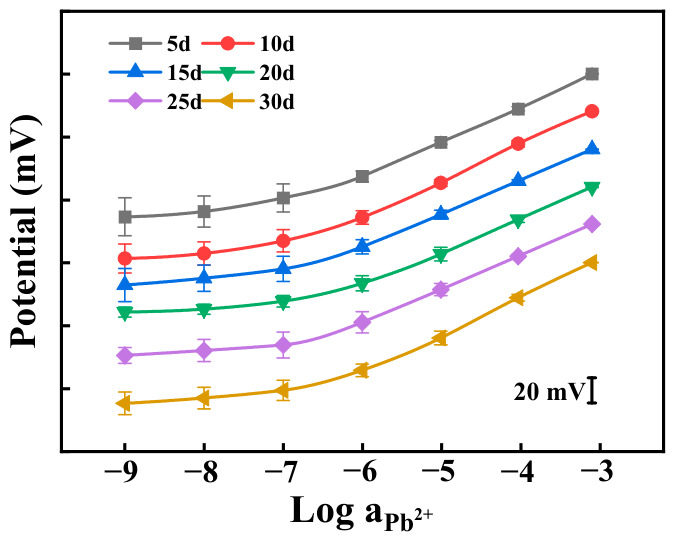
Calibration curves of the Pb^2+^-ISμE for different days. Error bars represent the standard deviation of three measurements.

In this study, the lifetime of the proposed microelectrode was characterized by measuring the time-dependent changes in the response slope and detection limit. The potentiometric response was measured at certain intervals. The standard deviations were calculated based on measurements performed with three independent sensors. A 10^−5^ M Pb(NO_3_)_2_ solution was used for storing the electrodes when they were not tested. The time at which the slope exhibits no significant difference can be defined as the lifetime [29]. As shown in Figure 6, the Pb^2+^-ISμE maintains a Nernstian response in the concentration range of 1.0 × 10^−7^–1.0 × 10^−3^ M within 30 days. Only a 2.2% decline in the response slope is observed, which changes from 27.4 ± 0.7 mV/decade on day 5 to 26.8 ± 2.2 mV/decade on day 30 (Table 1). Meanwhile, the detection limit of the microelectrode slightly increases from 4.2 × 10^−8^ M to 8.6 × 10^−8^ M over the 30-day period. These results indicate that the proposed Pb^2+^-ISμE can be used for 30 days. It should be noted that a slow decreasing trend is also observed. With extended usage time, the slope is expected to degrade.

The standard deviation of the detection limit on day 5 is relatively small, indicating good repeatability of the electrode in its initial state. As the usage time increases, the standard deviations become larger. Such an increase indicates a deterioration in the electrode’s response stability at low concentrations over time, which is primarily ascribed to the loss of components from the sensitive membrane. Although the detection limit remains below the lowest concentration of the linear range within 30 days, an increase in the detection limit may lead to larger errors when measuring at low concentrations. As the detection limit continues to increase, the linear range of the electrode will be compromised. Moreover, the noise of the microelectrode measured in 10^−5^ M Pb(NO_3_)_2_ (without stirring) increases from 0.1 mV to 0.2 mV in 30 days, which is attributed to the increase in electrode impedance over time.

The use of microelectrodes with a long lifetime can reduce costs and ensure the reliability of long-term measurements. Microelectrodes may remain functional for extended periods under proper storage conditions. In this work, we focus on the lifetime of ion-selective microelectrodes under continuous use in solutions. Table 2 summarizes the lifetimes of both the previously reported microelectrodes and the proposed Pb^2+^-ISμE. All lifetimes were evaluated in standard solutions. The proposed Pb^2+^-ISμE exhibits a longer lifetime than most ion-selective microelectrodes. This is mainly due to the thickness of the ISM, which not only ensures good mechanical stability of the ISM but also provides a larger amount of membrane components, thereby mitigating the negative effects of leaching of membrane components.

The lifetime of a microelectrode is determined primarily by the ISM type and the electrode configuration. Liquid membranes lack polymeric matrices to support membrane components, making them susceptible to leakage and ultimately leading to a sensor failure [22]. For polymeric membranes, although the use of polymeric matrix material can stabilize the membrane phase, the slow leaching of membrane components may lead to a gradual deterioration in sensor performance. In recent years, solid-state ion-selective microelectrodes have been widely studied [21]. A commonly used approach for constructing such microelectrodes is to directly coat the ISM onto a microscale conductive substrate with an SC layer [12]. However, due to the thinness of the ISM, a water layer easily forms between the membrane and the SC layer, resulting in a short electrode lifetime. An alternative approach is to fix the ISM at the micropipette tip and contact it with the conductive substrate containing the SC layer [30]. However, the conductive substrate is located at the tip of the micropipette, resulting in a typically large tip size for this type of electrode.

Increasing the thickness of the polymeric sensing membrane offers a simple route to fabricating microelectrodes with a long lifetime. However, a thick membrane also leads to a slow response. In contrast, microelectrodes with a thin membrane exhibit a fast response. However, such membranes contain a smaller total amount of active components, and the loss of these components significantly shortens the electrode lifetime. Therefore, microelectrode design must balance the trade-off between lifetime and response speed.

**Table 2:** Comparison of lifetime of the ion-selective microelectrodes reported in the literature and the proposed Pb^2+^-ISμE.

Analyte	Type	ISM	Tip Diameter (μm)	ISM Thickness (μm)	Slope (mV Decades^−1^)	Detection Limit (M)	Lifetime	Reference
Mg^2+^	LC ^1^	Liquid membrane (*o*-NPOE)	1	250	29.4	5.0 × 10^−5^	7 days	[31]
K^+^	LC	Liquid membrane (DNB ^2^)	10–20	1000–2000	55.6	-	7 days	[32]
Pb^2+^	LC	Liquid membrane (*o*-NPOE)	5	-	-	3.2 × 10^−7^	few hours	[30]
CO_3_^2−^	LC	Liquid membrane (DOA ^3^)	10–15	200	−25.0–−29.0	2.5 × 10^−5^	2–6 h	[13]
CO_3_^2−^	LC	Liquid membrane (*o*-NPOE)	10–15	400	−27.0–−30.0	5.0 × 10^−6^	3–5 days	[13]
NO_3_^−^	LC	Polymeric membrane (PVC, *o*-NPOE)	1–2	100–400	−44.9	7.9 × 10^−6^	3 days	[33]
CO_3_^2−^	LC	Polymeric membrane (PVC, DOA)	20	100	−27.1	2.0 × 10^−6^	5–7 days	[14]
H^+^	LC	Polymeric membrane (PVC, *o*-NPOE)	1	100–300	57.5	-	4 days	[34]
Ca^2+^	SC ^4^	Polymeric membrane (PVC, *o*-NPOE)	80	50–100	30.8	1.2 × 10^−7^	7 days	[9]
NH_4_^+^	SC	Polymeric membrane (PVC-COOH ^5^, DOS ^6^)	-	-	58.2	7.5 × 10^−6^	15 days	[35]
NH_4_^+^	SC	Solid membrane (CuHCF ^7^)	22.2	7.63	59.0	6.2 × 10^−7^	9 days	[20]
Pb^2+^	SC	Polymeric membrane (PVC, *o*-NPOE)	14	80	28.1	6.4 × 10^−10^	3 days	[12]
Pb^2+^	SC	Polymeric membrane (PVC, *o*-NPOE)	15	60	27.1	6.3 × 10^−9^	10 days	[30]
Cu^2+^	SC	Polymeric membrane (PVC, *o*-NPOE)	21.3	80	34.8	5.8 × 10^−9^	6 days	[36]
Pb^2+^	LC	Polymeric membrane (PVC, *o*-NPOE)	5	600	27.4	4.2 × 10^−8^	30 days	This work

^1^ Liquid-contact; ^2^ 1,2-dimethyl-3-nitrobenzene; ^3^ Bis(2-ethylhexyl)adipate; ^4^ Solid-contact; ^5^ Carboxylated polyvinyl chloride; ^6^ Bis(2-ethlhexyl) sebecate; ^7^ Copper hexacyanoferrate.

## 4. Conclusions

In this study, a liquid-contact ion-selective microelectrode with an extended lifetime was fabricated by increasing the thickness of the polymeric sensing membrane. The shape of the microelectrode tip and the amount of membrane solution were optimized. A microelectrode with both structural integrity and good performance can be obtained by using a micropipette with a tapered tip and 0.25 μL of membrane solution. The proposed Pb^2+^-ISμE exhibited a Nernstian response slope of 27.4 ± 0.7 mV decade^−1^ over the concentration range from 1.0 × 10^−7^ to 1.0 × 10^−3^ M, with a detection limit of 4.2 × 10^−8^ M. Notably, the response slope decreased by only 2.2% in 30 days. The proposed microelectrodes could be more rugged and durable than thin-membrane designs, making them more favorable for in situ environmental monitoring. The proposed strategy is configuration-independent and can be easily applied to develop other long-lifetime microelectrodes. For ionophores that are highly sensitive to membrane composition or matrix effects, the membrane-thickening strategy may require re-optimization of the filling solution parameters. It should be noted that the long lifetime of the proposed microelectrode was based on measurements in standard solutions and has not yet been validated in real samples of complex matrices, where membrane degradation can occur faster, resulting in a shorter sensor lifetime. Future research should focus on developing microelectrodes with a long lifetime in real application scenarios.

## Figures and Tables

**Table 1:** Response slope and detection limit of the Pb^2+^-ISμE for different days.

Day	Slope (mV/Decade)	Detection Limit (×10^−8^ M)
5	27.4 ± 0.7	4.2 ± 0.6
10	27.6 ± 1.0	4.8 ± 2.1
15	27.6 ± 1.5	6.3 ± 3.4
20	27.4 ± 1.8	6.5 ± 3.1
25	26.6 ± 1.1	8.3 ± 3.1
30	26.8 ± 2.2	8.6 ± 2.7

## Data Availability

Data will be made available on request.
